# Comparison among the Quantification of Bacterial Pathogens by qPCR, dPCR, and Cultural Methods

**DOI:** 10.3389/fmicb.2017.01174

**Published:** 2017-06-28

**Authors:** Matteo Ricchi, Cristina Bertasio, Maria B. Boniotti, Nadia Vicari, Simone Russo, Michela Tilola, Marco A. Bellotti, Barbara Bertasi

**Affiliations:** ^1^Istituto Zooprofilattico Sperimentale della Lombardia e dell'Emilia Romagna “Bruno Ubertini,” National Reference Centre for ParatuberculosisPodenzano, Italy; ^2^Istituto Zooprofilattico Sperimentale della Lombardia e dell'Emilia Romagna “Bruno Ubertini,” National Reference Centre for Tuberculosis from M. bovisBrescia, Italy; ^3^Istituto Zooprofilattico Sperimentale della Lombardia e dell'Emilia Romagna, National Reference Laboratory for Tularemia, Istituto Zooprofilattico Sperimentale della Lombardia e dell'Emilia Romagna “Bruno Ubertini”Pavia, Italy; ^4^Istituto Zooprofilattico Sperimentale della Lombardia e dell'Emilia Romagna “Bruno Ubertini,” Reparto Tecnologie Acidi Nucleici Applicate Agli AlimentiBrescia, Italy

**Keywords:** dPCR, qPCR, bacteria, quantification, pathogens

## Abstract

The demand for rapid methods for the quantification of pathogens is increasing. Among these methods, those based on nucleic acids amplification (quantitative PCRs) are the most widespread worldwide. Together with the qPCR, a new approach named digital PCR (dPCR), has rapidly gained importance. The aim of our study was to compare the results obtained using two different dPCR systems and one qPCR in the quantification of three different bacterial pathogens: *Listeria monocytogenes, Francisella tularensis*, and *Mycobacterium avium* subsp. *paratuberculosis*. For this purpose, three pre-existing qPCRs were used, while the same primers and probes, as well as PCR conditions, were transferred to two different dPCR systems: the QX200 (Bio-Rad) and the Quant Studio 3D (Applied Biosystems). The limits of detection and limits of quantification for all pathogens, and all PCR approaches applied, were determined using genomic pure DNAs. The quantification of unknown decimal suspensions of the three bacteria obtained by the three different PCR approaches was compared through the Linear Regression and Bland and Altman analyses. Our results suggest that, both dPCRs are able to quantify the same amount of bacteria, while the comparison among dPCRs and qPCRs, showed both over and under-estimation of the bacteria present in the unknown suspensions. Our results showed qPCR over-estimated the amount of *M. avium* subsp. *paratuberculosis* and *F. tularensis* cells. On the contrary, qPCR, compared to QX200 dPCR, under-estimated the amount of *L. monocytogenes* cells. However, the maximum difference among PCRs approaches was <0.5 Log_10_, while cultural methods underestimated the number of bacteria by one to two Log_10_ for *Francisella tularensis* and *Mycobacterium avium* subsp. *paratuberculosis*. On the other hand, cultural and PCRs methods quantified the same amount of bacteria for *L. monocytogenes*, suggesting for this last pathogen, PCRs approaches can be considered as a valid alternative to the cultural ones.

## Introduction

The advent of molecular tools for the rapid detection of bacterial pathogens has profoundly changed diagnosis methods both in term of rapidity and cost effectiveness (Law et al., 2014). In this regard, an impressive amount of new protocols based on molecular amplification approaches have been developed. These methods are considered alternative or, in some cases, complementary to the classical methods (i.e., cultures).

One of the most popular molecular tools is the polymerase chain reaction (PCR) and, more recently, the quantitative PCR (qPCR), has become one of the most used methodologies for the detection of a wide range of pathogens, including viruses, bacteria, and parasites (Law et al., 2014; Sloots et al., 2015). In addition, this last application also permits the absolute quantification of pathogens present in a given amount of sample. For this purpose, in order to generate calibration curves, standards, containing known amounts of plasmids, genomic DNAs or other nucleic acid molecules (NAs) are run, in parallel with the unknown samples. According to semi-log regression model, it is possible to establish the amount of nucleic acid in unknown specimens (Anonymous, 2012). Based on this feature, and taking into account the efficiency and the dilution steps required for the NA extraction and analysis, it is possible to determine the absolute number of pathogens in the unknown samples (Ricchi et al., 2016).

Another recent PCR-based approach, named digital PCR (dPCR), permits the quantification of the NA present in PCR tubes. Conversely to qPCR, this approach does not require calibration curves for quantification, but it is based on sample partitioning, so that individual nucleic acid molecules are amplified by an end-point PCR and positive partitions can be an estimate of target concentration through Poisson distribution (Hudecova, 2015). Finally, dPCR is considered more robust, reliable and less sensitive to inhibitors than qPCR (Devonshire et al., 2015).

The aim of our study was to compare the quantifications performed by both PCR approaches (qPCR and dPCR) in the enumeration of three pathogen bacteria suspended in unknown water suspensions. The bacteria considered in the study were: *Listeria monocytogenes, Francisella tularensis*, and *Mycobacterium avium* subsp. *paratuberculosis* (MAP).

These bacteria grow in solid cultures at different rates, *L. monocytogenes* being the fastest (approximately 1 day), then *F. tularensis* (approximately 3 days) and MAP the slowest (approximately 30–50 days) and since the different growing rates can have an impact in the quantification done by cultural plating (Kralik et al., 2012), the amount of bacteria present in unknown suspensions was quantified by pathogen-specific qPCRs, by two different dPCRs systems, the QX200™ Droplet Digital™ PCR System (Bio-Rad, Berkeley, USA) and the QuantStudio™ 3D digital PCR system (QS3D; Applied Biosystems). The obtained results were also compared with each other and with the relative cultural assays, with the method based on the measure of absorbance at 600 nm and, but only for MAP, direct microbial count (Bürker chamber).

The three bacteria were selected as representative of mycobacteria genus, gram positive, and gram negative bacteria. In more detail, *L. monocytogenes* is a gram positive bacterium responsible for listeriosis, a foodborne illness which predominantly affects pregnant women, elderly people, neonates, and adults with impaired immune systems. Since this pathogen is ubiquitarious and it is very difficult to guarantee its absence in many ready to eat products; the European regulation 2073/2005, based on a risk assessment approach, stated the maximum amount that should be present in food, (Anonymous, 2005).

*F. tularensis* is a gram-negative, facultative intracellular bacteria causing tularaemia, a zoonotic disease that could be fatal to humans and animals. Tularaemia can be transmitted with infected animals or ticks bites, via inhalation or ingestion of contaminated aerosol or food. Due to low infectious dose (as few as 10 microorganisms) and airborne transmission, *Francisella* is currently classified as a category a biological agent (Carvalho et al., 2014).

*M. avium* subsp. *paratuberculosis* (MAP) is the etiological agent of paratuberculosis (Johne's Disease), a chronic enteritis affecting wild and domestic ruminants. A possible zoonotic role of MAP in the developing of Crohn' disease is still debated in literature (Chiodini et al., 2012).

Finally, in order to ascertain the possibility of using molecular methods for the quantification of bacterial pathogens, for only *L. monocytogenes*, the level of uncertainty associated to the quantification of each method was evaluated.

## Materials and methods

### Quantitative PCRs and digital PCRs

#### qPCRs

The absolute number of bacterial quantity through qPCR was determined using a calibration curve generated with genomic DNA.

The MAP genomic DNA used to build the standards for the absolute quantification of MAP was kindly provided by Dr. Plain from University of Sidney; the *L. monocytogenes* genomic DNA and the *F. tularensis* genomic DNA were extracted from ATCC 13922 and ATCC 6223 strains, respectively. *L. monocytogenes* and *F. tularensis* genomic DNA was initially quantified by QuantiFluor® dsDNA System (Promega, Milan, Italy).

MAP quantification by qPCR targeting the f57 region (f57-qPCR) was performed in 20 μL (final volume) containing 2x Taqman Universal PCR mastermix (Applied Biosystems), 300 nM primers each and 150 nM Taqman TAMRA Probe (Ricchi et al., 2014, 2016) in a StepOne Plus system (Applied Biosystems). *L. monocytogenes* amplifications were carried out targeting the listeriolysin O (hlyA) gene using primers and probe already described (Traunsek et al., 2011). This last qPCR assay was performed with 2x Taqman Universal PCR mastermix (Applied Biosystems), 600 nM primers each and 200 nM Taqman MGB Probe in CFX96 (Bio-Rad) real-time PCR System. The thermal cycling conditions were: 50°C × 2 min, 95°C × 10 min, 45 cycles of 15 s at 95°C, and 1 min at 60°C. *F. tularensis* qPCRs were performed using primers and probe targeting the 23 kDa protein gene, as previously described (Versage et al., 2003). Amplification was carried out with 2x SsoAdvanced Universal Probes Supermix (Bio-Rad), 600 nM of each primer and 200 nM of Taqman Probe in CFX96 (Bio-Rad) real-time PCR System. The thermal cycling conditions were: 95°C × 2 min, 45 cycles of 5 s at 95°C, and 10 s at 60°C. For each run, at least one negative template control (NTC) was included. Cq determination was performed using the fit point method.

Quantitative PCRs were developed and validated according to the MIQE guidelines (Bustin et al., 2009). This information is available in the Data Sheet 1 (Supplementary Material 1) of the paper.

The qPCR information relative to the performance of the assays was already reported in the original papers or listed in the Data Sheet 1. For each reaction, five points of genomic DNA log-dilution were added in triplicate to each run and used as standards for the absolute bacterial quantification. This was evaluated considering the genome size and the amount of added DNA using a semi-log model based on an excel datasheet (available on request).

#### dPCRs

Digital PCR reactions were performed in two different systems: QX200™ Droplet Digital™ PCR System (Bio-Rad) and Applied Biosystems® QuantStudio™ 3D digital PCR system (QS3D), for all three bacteria. The reactions were carried out using the same primers and probes concentrations as for qPCRs. Results were expressed as number of bacterial cells per μL.

For Bio-Rad system, five μL of DNA were added to 10 μL of 2X ddPCR™ Supermix for Probes (No dUTP; Bio-Rad), to the specific forward/reverse primers and probe, in a final volume of 20 μL. Droplets were generated using a droplet generator cartridge in a QX200™ Droplet Generator (Bio-Rad), according to the manufacturer's instructions. The droplets emulsion was then loaded into an ABI thermal cycler (Applied Biosystem). Amplification conditions for all three targets were: 10 min at 95°C for enzyme activation, followed by 40 cycles of a two-step thermal profile of 30 s at 94°C (denaturation), 1 min at 60°C (annealing/extension) with a reduced ramp rate of 2°C/s, and a final 10 min vation step at 98°C. Plates were then transferred to a QX200™ Droplet Reader (Bio-Rad). For each run, at least one No Template Control (NTC) was included. All of the thresholds were set up manually to allow the discrimination between positive and negative droplets: two positive droplets were enough to determine a sample as positive, and only the reactions with more than 10,000 accepted droplets were used for analysis. All NTCs resulted negative. In the present study, the number of accepted droplets ranged from 11,439 to 18,415, with a mean of 15,376. Absolute quantification of PCR targets was performed using QuantaSoft™ software version 1.7.4.0917 (Bio-Rad). The mean number of copies per partition (λ), the number of estimated copies per total reaction volume (20 μL), the number of estimated copies per mean effective reaction size were calculated (Pavšič et al., 2016). The mean λ-value was also determined for each sample dilution. The sample concentration expressed as copies per μL was estimated both on the basis of total reaction volume and effective reaction size (Pavšič et al., 2016). On the basis of the droplets volume of 0.85 nL used for QX200 system, the mean effective reaction size was estimated as 12.99 (±1.68) μL. Students' tests were used to determine the statistical significance of the sample concentration values, considering the total reaction volume and the effective reaction size.

For the Applied Biosystems QuantStudio™ 3D digital PCR system (QS3D), the reactions were prepared in a final volume of 16 μL, composed by 10.7 μL of Mastermix QuantStudio® 3D Digital PCR (Applied Biosystems, Milan, Italy) and 5.3 μL of DNA at different dilutions; primers and probes were used at the concentrations described for qPCR. Results were expressed as bacterial cells per μL. The reaction mix (15 μL out of 16 μL) was loaded onto the QuantStudio 3D digital PCR chips by using QuantStudio 3D digital PCR chip loader. The amplification conditions for all bacteria were: 96°C × 10 min; 60°C × 2 min, 98°C × 30 s, 39 cycles; 60°C × 2 min. The amplifications were performed in a Proflex™ 2x Flat PCR System. The chips were transferred to a QS3D Instrument for imaging. Data elaboration was executed using the cloud-based QuantStudio 3D Analysis Suite software (version 3.0.03) in the absolute quantification module maintaining automatic settings. For each run, at least one NTC was included. The quality threshold was set at the default value of 0.5, to define the accepted wells and ranged from 10,518 to 18,608 with a mean of 16,838.

Also for QS3D dPCR, the mean number of copies per partition (λ) and the number of estimated copies per total reaction volume (15 μL) for the unknown samples were calculated.

The information relative to the dPCRs, as suggested by MIQE guideline for digital PCR (Huggett et al., 2013), are listed in the Data Sheet 1 (Supplementary Material 1) of the present paper.

#### Preparation of unknown bacterial suspensions for the quantification

In order to minimize sources of variability due to different efficiencies of the method chosen during DNA extraction and purification (Devonshire et al., 2015), 5 μl of unknown suspensions were used as templates for qPCRs and dPCRs without any other set up processing (no DNA extraction kits were used). Results were expressed as the number of bacterial cells per μL.

#### *Mycobacterium avium* subsp. *paratuberculosis*

A MAP reference strain (ATCC 19698) and two MAP field isolates (IZSLER 623/15 and 22/16) were used. MAP suspensions were prepared according to Logar et al. (2012) and Plain et al. (2014). Briefly, colonies from solid cultures were suspended in distilled water with glass beads (diameter ca. 5 mm) and vortexed for 45 s. The optical density at 600 nM was adjusted to be around 0.7. The suspensions were forced through a syringe (needle 26 G) three times and filtered through a sterile 5 μm filter and then examined in a Bürker chamber to count the number of MAP cells (expressed as MAP cells per mL of suspension). The initial suspensions were 10-fold serially diluted in 1 mL of distilled water in tubes with glass beads (diameter ca. 5 mm) and vortexed for 20 s between dilution steps. One hundred microliters of each dilution were streaked in duplicate onto Petri plates (diameter 9 cm) of Herrold's Egg Yolk Agar with Mycobactin J 2 mg/L (HEYM) for the determination of MAP concentrations expressed as CFU per μL. Plates were incubated at 37°C and the number of CFU was counted approximately 80 days after the inoculum. For the initial suspensions, the optical density at 600 nm was also recorded three times in BioPhotometer® (Eppendorf, Milan, Italy).

The remaining parts of the suspensions (approximately 850 μL) were then submitted to bead beating (Tissue Lyser II, Qiagen, Milan, Italy) for 10 min at 30 Hz in the presence of 300 mg of 150–212 μm-diameter acid-washed glass beads (Sigma Aldrich, Milan, Italy). The suspensions were then heated at 100°C for 20 min, centrifuged at 16,000 g for 5 min and 5 μl of the supernatant were processed by qPCR and dPCRs.

#### Listeria monocytogenes

Regarding *L. monocytogenes*, three field isolates, IZSLER 133-1/2016, 129516-1/2016, and 424965-2/2016 were used. The strains were prepared as described for MAP isolates and the absorbance read at 600 nM by DU 800 spectrophotometer (Beckman Coulter). Burker chamber counting was not performed. Plate counting was performed on blood agar as described in ISO 11290-2 (Anonymous, 1998), incubated at 37°C for 24 h. The remaining parts of the suspensions were then heated at 100°C for 20 min and centrifuged at 16,000 g for 5 min. Five microliters were used as the template for qPCR and dPCRs.

#### Francisella tularensis

A *F. tularensis* reference strain (ATCC 6223) and two field isolates (IZSLER 42055/2008 and 318595/2009) were used. *F. tularensis* suspensions were prepared as previously reported for MAP and *L. monocytogenes*, without glass beads treatment between each dilution. The optical density at 600 nm was recorded but counting through Burker chamber was not performed. Fifty microliters of each dilution were streaked in duplicate onto Petri plates (diameter 9 cm) on Cysteine Heart Agar (CHA) to determine *F. tularensis* concentrations expressed as CFU per μL. Plates were incubated at 37°C and examined approximately 5 days after the inoculum in order to record the number of CFU.

The remaining parts of the suspensions were then heated at 100°C for 20 min and centrifuged at 16,000 *g* for 5 min. Five microliters were used as template for qPCR and dPCRs.

#### Limit of detection (LOD) and limit of quantification (LOQ) of the PCR assays

The LOD and LOQ for qPCRs and dPCRs were evaluated using genomic DNA for each bacterium. For qPCRs, the limits of detection is represented by 95% of a positive call, herein defined as LOD_95%_ were evaluated using genomic DNAs. According to Pavsic (Pavšič et al., 2016), five replicates in two independent runs of serially diluted genomic DNA were analyzed (10 replicates in total), and logit functions instead of the probit functions were used (Burns and Valdivia, 2008). Notably, this approach was also recommended by the OIE Manual of Diagnostic Tests and Vaccines for Terrestrial Animals (Anonymous, 2014). Results were expressed as Log_10_ fg per reaction.

As stated in the above cited paper (Pavšič et al., 2016), the limit of quantification (LOQ) for qPCRs was fixed at the lowest concentration where all replicates were positive and the coefficient of variation was up to 25%. The same limits were used for LOQ determination for both dPCRs systems (Pavšič et al., 2016), while the LOD for dPCRs was fixed at the lowest concentration where all replicates gave a positive results (Pavšič et al., 2016). Results were expressed as number of cells per μL. The variability of dPCRs was estimated evaluating the intra run and the inter run variations expressed as coefficients of variations. In more detail, intra run variability was evaluated analysing five dilutions tested in five replicates of genomic standard DNAs; the inter run variability was evaluated analysing the same five dilutions in five replicates tested in two independent runs.

#### Statistical analysis for the comparison between PCR approaches in unknown samples

Agreement and linear regression between the different PCR methods were evaluated, after Log_10_ data transformation, according to the statistical approaches proposed by Bland and Altman (Bland and Altman, 1986). Normality of differences between the quantity obtained by the three PCR methodologies for unknown samples was assessed by Shapiro-Wilk test (STATA 12, Texas, USA). Graphs were generated using MATLAB software developed by MathWorks (Massachusetts, USA). The level of uncertainty associated to the quantification for *L. monocyotogenes* was evaluated as reported in the ISO/TS 19036 (Anonymous, 2009) for all the methods. In particular, the concentration of *L. monocytogenes* cells (values around the LOQ) were Log_10_ transformed and the expanded uncertainty was calculated with the formula:

$$\begin{array}{l} U=2\sqrt{{S_{R}}^{2}+\frac{0.18861}{\sum c}} \end{array}$$

where 0.18861/Σ*c* is the variance component due to the Poisson distribution, in which Σ*c* is the sum of the total numbers of colonies/cells counted. Notably the factor 2 gives a level of confidence of 95%.

S_*R*_ is the reproducibility standard deviation calculated with the formula:

$$\begin{array}{l} S_{R}=\sqrt{\frac{1}{n}\overset{n}{\underset{i=1}{\sum }}\frac{{\left(y_{iA}-y_{iB}\right)}^{2}}{2}} \end{array}$$

where *y*_*ij*_ are the Log_10_ transformed data, i is the index of the sample, *i* = 10 to *n* (*n* ≥ 10); j is the index of reproducibility condition, *j* = A or B.

## Results

### Determination of qPCRs and dPCRs performance

**Figure 1:**
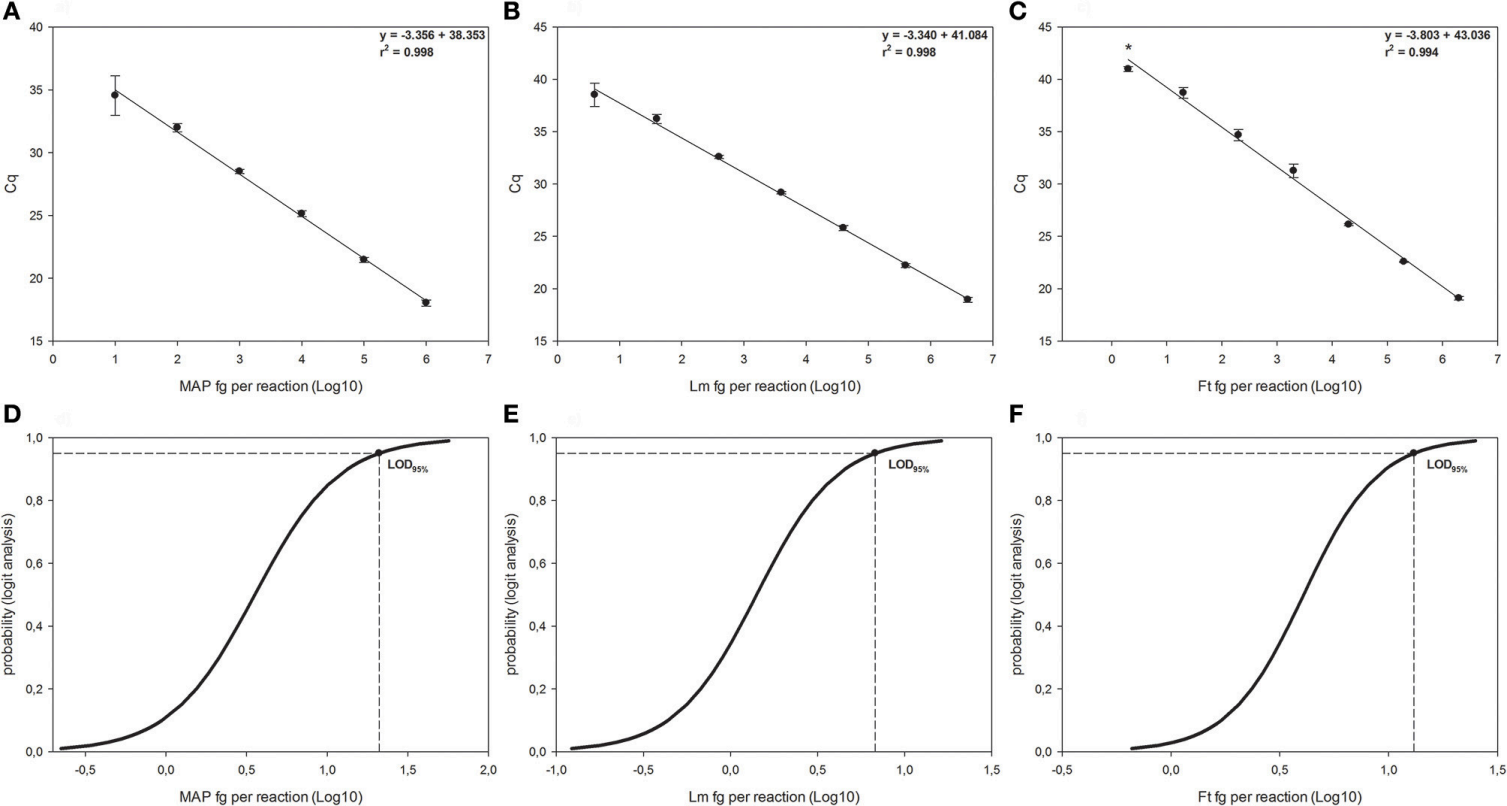
Linear regressions and Logit analysis plots of the three qPCRs used in the study. Panels **(A,D)** are referred to *Mycobacterium avium* subsp. *paratuberculosis* (MAP), panels **(B,E)** are referred to *Listeria monocytogenes* (Lm) and panels **(C,F)** to *Francisella tularensis* (Ft). The number of replicates used for the evaluation of the panels **(A–C)** is reported in material and method section. In the right side of each graph are reported the first-grade equation as well as the *r*^2^ values. ^*^Indicates 2/10 replicates were positive. Bars represent standard deviation. Panels **(D–F)** show the LOD_95%_ for each qPCR, which are the minimum amounts of DNA detectable with a 95% probability.

The LOD and LOQ of the three different qPCRs, as well as those of the two dPCR systems, were reported in Figure 1 and Table 1, respectively.

**Table 1:** Performances of dPCR systems used in the study for detection and quantification of *M. avium* subsp. *paratuberculosis, L. monocytogenes*, and *F. tularensis*.

	***M. avium* subsp. *paratuberculosis***	***L. monocytogenes***	***F. tularensis***
**Key Characteristics of dPCR Assay (QX200)**			
Dynamic Range (fg per μL)^*^	10^2^–10^5^	10^1^–10^4^	10^0^–10^3^
Dynamic Range (Log_10_ copies/μL)^◦^	1–4	1–4	0–3
LOD—n^◦^ of target copies/μL	18.120 ± 2.946^#^	10.040 ± 1.391^#^	2.204 ± 0.847
LOQ—n^◦^ of target copies/μL	18.120 ± 2.946^#^	10.040 ± 1.391^#^	27.240 ± 3.295
Intra-run Variation: Range of Coefficients of variation (five dilutions tested in five replicates)	±0.9 to ±15.7%	±3.3 to ±14.1%	±4.4 to ±34.0%
Inter-run Variation: Range of coefficient of variation (five dilutions tested five times in two independent replicate runs)	±6.8 to ±11.9%	±2.0 to ±5.6%	±1.0 to ±25.4%
**Key Characteristics of dPCR Assay (Quant Studio 3D)**			
Dynamic Range (fg per μL)^*^	10^1^–10^5^	10^1^–10^4^	10^0^–10^3^
Dynamic Range (Log_10_ copies/μL)^◦^	0–4	1–4	0–3
LOD—n^◦^ of target copies/μL	2.097 ± 1.345	13.813 ± 2.858^#^	3.367 ± 1.547
LOQ—n^◦^ of target copies/μL	17.769 ± 3.364	13.813 ± 2.858^#^	31.539 ± 4.758
Intra-run Variation: Range of Coefficients of variation (from five dilutions tested in five replicates)	±3.4 to ±64.3%	±5.9 to ±34.6%	±4.7 to ±39.1%
Inter-run Variation: Range of coefficient of variation (five dilutions tested five times in two independent replicate runs)	±6.7 to ±22.6%	±3.1 to ±8.82%	±2.1 to ±8.4%

* *These ranges were determined considering the positive signals at LOD level obtained with the same genomic DNA standards used for evaluate the performances of qPCRs*.

◦ *These ranges were determined considering the LOD*.

# *The value is identical for LOD and LOQ because all replicates were positive and the coefficient of variation is lower than 25%; further details in the material and method section*.

For the f-57 qPCR relative to *M. avium* subsp. *paratuberculosis* (see Figures 1A,D), the LOD_95%_ was 1.322 Log_10_ fg per reaction (95% CI 0.924–2.739), corresponding to 3.964 genomic equivalents MAP cells (20.989 fg per reaction divided by 5.295 fg, which is the weight of a single MAP's genome). The LOQ was estimated around 100 fg per reaction, corresponding to 1.889 × 10^1^ genomic equivalent (coefficient of variation 23.419%). The efficiency of the reaction was 98.827 ± 7.146%.

The qPCR for *L. monocytogenes* (see Figures 1B,E) showed a LOD_95%_ of 0.831 Log_10_ fg per reaction (95% CI 0.460–2.104), corresponding to 2.060 × 10^0^ genomic equivalents *L. monocytogenes* cells (6.776 fg per reaction divided by 3.289 fg, which is the weight of a single *L. monocytogenes* genome). The LOQ was fixed at 40 fg per reaction, corresponding to 1.216 × 10^1^ genomic equivalent (coefficient of variation 13.296%), being the concentration below (4 fg per reaction) with a coefficient of variation over 25% (27.265%). The efficiency of the reaction was 98.915 ± 5.226%.

Finally, for *F. tularensis* qPCR (see Figures 1C,F), the LOD_95%_ was 1.116 Log_10_ fg per reaction (95% CI 0.729–3.638), corresponding to 6.382 genomic equivalents *F. tularensis* cells (13.062 fg per reaction divided by 2.047 fg, which is the weight of single *F. tularensis*'s genome). The LOQ was 200 fg per reaction, corresponding to 9.772 × 10^1^ genomic equivalents *F. tularensis* cells (coefficient of variation 4.973%) and the efficiency of the reaction was 80.181 ± 5.781%.

The dynamic range of the reactions varied between one to six Log_10_ fg per reaction for MAP and between approximately one to seven Log_10_ fg per reaction for *L. monocytogenes* and *F. tularensis*.

The performance of the dPCRs are shown in Table 1. In order to avoid further variability due to the different setup between different PCR systems and approaches (digital or quantitative), primers and probes were used at the same concentration as for qPCRs. As expected, the dynamic ranges observed were lower than those obtained for the corresponding qPCRs for all bacteria; in fact, for *L. monocytogenes* target DNA over than 10^4^ fg per μL, both dPCR systems were saturated and it was not possible to obtain any quantification. The saturation amount was lower for *F. tularensis* (10^3^ fg per μL), while it was higher for MAP (10^5^ fg per μL).

Contrary to the observations carried out for qPCRs, the QX200 system for MAP and both dPCR systems for *L. monocytogenes* showed LOD and LOQ values overlapped each other, while for the rest of assays, the LOQ values were approximately one Log_10_ higher than LOD (Table 1).

The intra run variability (coefficients of variation) obtained from five dilutions, tested in five replicates, ranged from 0.9 to 34.0%, for the QX200 system, and from 3.4 to 64.3% for the QS3D. As expected, we observed an increased variability at the lowest DNA concentrations.

The inter run variability ranged from 1.0 to 25.4% for the QX200 and from 2.1 to 22.6% for the QS3D dPCR. These values were lower than those observed for the intra run, and, in this case, we found this variability increased according to the concentration of tested DNA.

### Evaluation of the number of bacteria in unknown suspensions by PCR approaches

The quantification results obtained for the unknown suspensions at different dilutions, for the three different PCRs (one qPCR and two dPCRs), are shown in Tables 2–**4**. All the NTCs were negative. For the QX200 system, sample concentrations, determined on the basis of total reaction volume and effective reaction size, did not show any statistical difference (*p* < 0.05). Therefore, we decided to use concentration values estimated considering the total reaction volume.

**Table 2:** Experimental output of MAP cells obtained by f57-qPCR, f57- QX 200 dPCR and f57- QuantStudio 3D dPCR.

		**qPCR^a^**			**dPCR QX 200^b^**				**dPCR QuantStudio 3D^b^**			
	**Dilution**	**Mean**	**SD**	**Signal ratio^c^**	**Mean**	**SD**	**Mean λ**	**Signal ratio^c^**	**Mean**	**SD**	**Mean λ**	**Signal ratio^c^**
ATCC 19698	−1	3.62 × 10^4^	1.33 × 10^4^	12/12	1.3 × 10^4^	7.92 × 10^2^	2.76	2/4	Saturated	Saturated		/
	−2	2.60 × 10^3^	1.06 × 10^3^	12/12	1.54 × 10^3^	7.82 × 10^1^	0.32	4/4	5.00 × 10^2^	3.46 × 10^1^	0.38	2/2
	−3	2.68 × 10^2^	1.43 × 10^2^	12/12	1.70 × 10^2^	1.31 × 10^1^	0.04	4/4	1.49 × 10^2^	3.12 × 10^1^	0.04	2/2
	−4	3.66 × 10^1^	2.19 × 10^1^	12/12	1.31 × 10^1^	2.66 × 10^0^	0.00	4/4	1.22 × 10^1^		0.02	1/2
	−5	6.41 × 10^0^	4.78 × 10^0^	8/12	2.16 × 10^0^	5.91 × 10^−1^	0.00	4/4	2.01 × 10^0^	8.77 × 10^−1^	0.00	2/2
	−6	0	0		1.09 × 10^0^	4.03 × 10^−1^	0.00	4/4	1.72 × 10^0^	0.95 × 10^−1^	0.00	2/2
	−7	0	0		6.10 × 10^−1^	7.57 × 10^−1^	0.00	2/4	4.89 × 10^−1^	0.30 × 10^−1^	0.00	2/2
IZSLER 623/15	−1	4.98 × 10^4^	1.37 × 10^4^	12/12	2.21 × 10^4^	1.14 × 10^4^	5.74	4/4	Saturated	Saturated		/
	−2	4.29 × 10^3^	1.20 × 10^3^	12/12	2.00 × 10^3^	2.09 × 10^2^	0.44	4/4	2.25 × 10^3^	1.88 × 10^2^	0.57	2/2
	−3	5.09 × 10^2^	1.41 × 10^2^	12/12	1.85 × 10^2^	1.37 × 10^1^	0.04	4/4	1.83 × 10^2^	3.05 × 10^1^	0.05	2/2
	−4	5.46 × 10^1^	1.75 × 10^1^	12/12	1.48 × 10^1^	1.46 × 10^0^	0.00	4/4	1.72 × 10^1^	7.47 × 10^0^	0.00	2/2
	−5	7.02 × 10^0^	3.38 × 10^0^	12/12	2.47 × 10^0^	4.82 × 10^−1^	0.00	4/4	1.43 × 10^0^	1.03 × 10^0^	0.00	2/2
	−6	2.44 × 10^0^	1.83 × 10^0^	10/12	4.93 × 10^−1^	3.03 × 10^−1^	0.00	3/4	2.50 × 10^0^	2.78 × 10^0^	0.00	1/2
	−7	8.03 × 10^−1^	6.64 × 10^−1^	3/12	2.70 × 10^−1^	3.77 × 10^−1^	0.00	1/4	5.23 × 10^−1^	4.29 × 10^−1^	0.00	2/2
IZSLER 22/16	−1	2.17 × 10^4^	8.31 × 10^3^	12/12	1.62 × 10^4^	3.67 × 10^3^	2.77	4/4	Saturated	Saturated		2/2
	−2	1.26 × 10^3^	2.55 × 10^2^	12/12	1.12 × 10^3^	4.59 × 10^1^	0.24	4/4	1.35 × 10^3^	4.27 × 10^1^	0.34	2/2
	−3	1.03 × 10^2^	1.99 × 10^1^	12/12	8.36 × 10^1^	5.36 × 10^0^	0.02	4/4	1.06 × 10^2^	3.96 × 10^−1^	0.03	2/2
	−4	1.21 × 10^1^	4.82 × 10^0^	12/12	1.10 × 10^1^	2.23 × 10^0^	0.00	4/4	9.47 × 10^0^	1.09 × 10^0^	0.00	2/2
	−5	1.57 × 10^0^	1.02 × 10^0^	12/12	1.93 × 10^0^	7.48 × 10^−1^	0.00	4/4	1.53 × 10^0^	2.14 × 10^−1^	0.00	2/2
	−6	8.55 × 10^−1^	6.50 × 10^−1^	10/12	3.50 × 10^−1^	2.95 × 10^−1^	0.00	3/4	4.80 × 10^−1^	3.42 × 10^−1^	0.00	2/2
	−7	3.07 × 10^−1^	7.25 × 10^−2^	4/12	4.20 × 10^1^	1.48 × 10^−1^	0.00	4/4	6.26 × 10^−1^	5.20 × 10^−1^	0.00	2/2

a *Number of MAP cells/μL in the pure cultures/suspensions evaluated by f57-qPCR. Data are shown as mean and standard deviation of four qPCR runs in triplicated*.

b *Number of MAP cells/μL in the pure cultures/suspensions evaluated by f57-dPCRs. Data are shown as mean and standard deviation of two dPCR runs in duplicated for QX200, and one dPCR run in duplicated for QuantStudio 3D*.

c *Number of positive or acceptable replicates/total number of replicates*.

#### *Mycobacterium avium* subsp. *paratuberculosis* (MAP)

**Figure 2:**
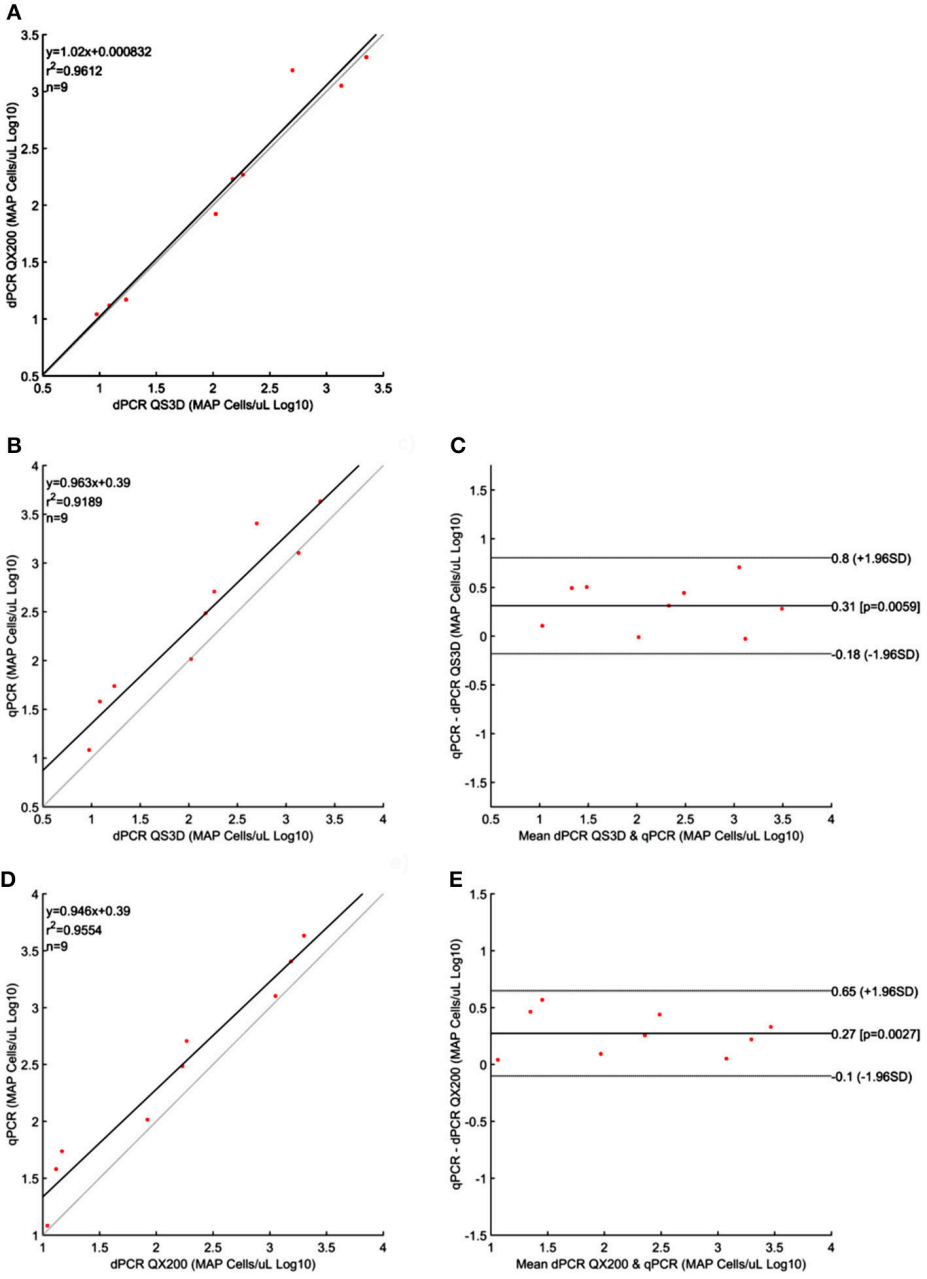
Linear regressions and Bland and Altman analyses of *Mycobacterium avium* subsp. *paratuberculosis* (MAP) cells in unknown samples obtained by dPCRs (QuantStudio 3D and QX200) and qPCR. **(A)** Linear regression between the two dPCRs; **(B)** Linear regression of dPCR QuantStudio 3D and qPCR; **(C)** Bland and Altman analysis of dPCR QuantStudio 3D and qPCR; **(D)** Linear regression of dPCR QX200 and qPCR; **(E)** Bland and Altman analysis of dPCR QX200 and qPCR. Bland and Altman analysis between the two dPCRs platform was not possible because the differences were not normally distributed. Data were reported as Log_10_ cells per μL. The gray lines in linear regression plots represent the ideal regression value.

For MAP, the quantification results showed approximately the same amount of Log_10_bacterial cells among different PCRs; however, for QS3D dPCR, the system was saturated at the highest concentration and it was not possible to count the number of copies present in the samples for all the analyzed strains. The regression lines and Bland and Altman analyses for MAP (Figure 2) were done by taking into account only the dilutions of unknown suspensions which varied from approximately 10^1^ MAP cells to 10^3^ MAP cells for all the strains tested. The lower limit was chosen because the LOQ for all PCRs was 10^1^ MAP cells per μL, while, over 10^3^ MAP cells, the QS3D dPCR was saturated. After Log_10_ transformation, the difference between the two dPCRs was not normally distributed (*p* = 0.002) and it was not possible to compare, by Bland and Altman analysis, the difference between the two dPCR systems. On the contrary, the Log_10_-difference between qPCR and QS3D dPCR, as well as that between qPCR and the QX200 dPCR, were distributed normally (*p* = 0.642 and *p* = 0.523, respectively). As expected, the linear regressions were well fitted for all comparisons, with *r*^2^-values between 0.919 and 0.961. Considering the Bland and Altman analysis, the bias for the agreement's range was 0.3 Log_10_ (0.27–0.31 Log_10_) for both analyses. The zero value fell inside the 95% CI of agreement limits, but *p*-values vs. zero were 0.006 and 0.003 for difference between qPCR and QS3D and qPCR and QX200, respectively. These results suggested the quantifications by qPCR were slightly higher than those obtained by both dPCR systems.

#### Listeria monocytogenes

**Figure 3:**
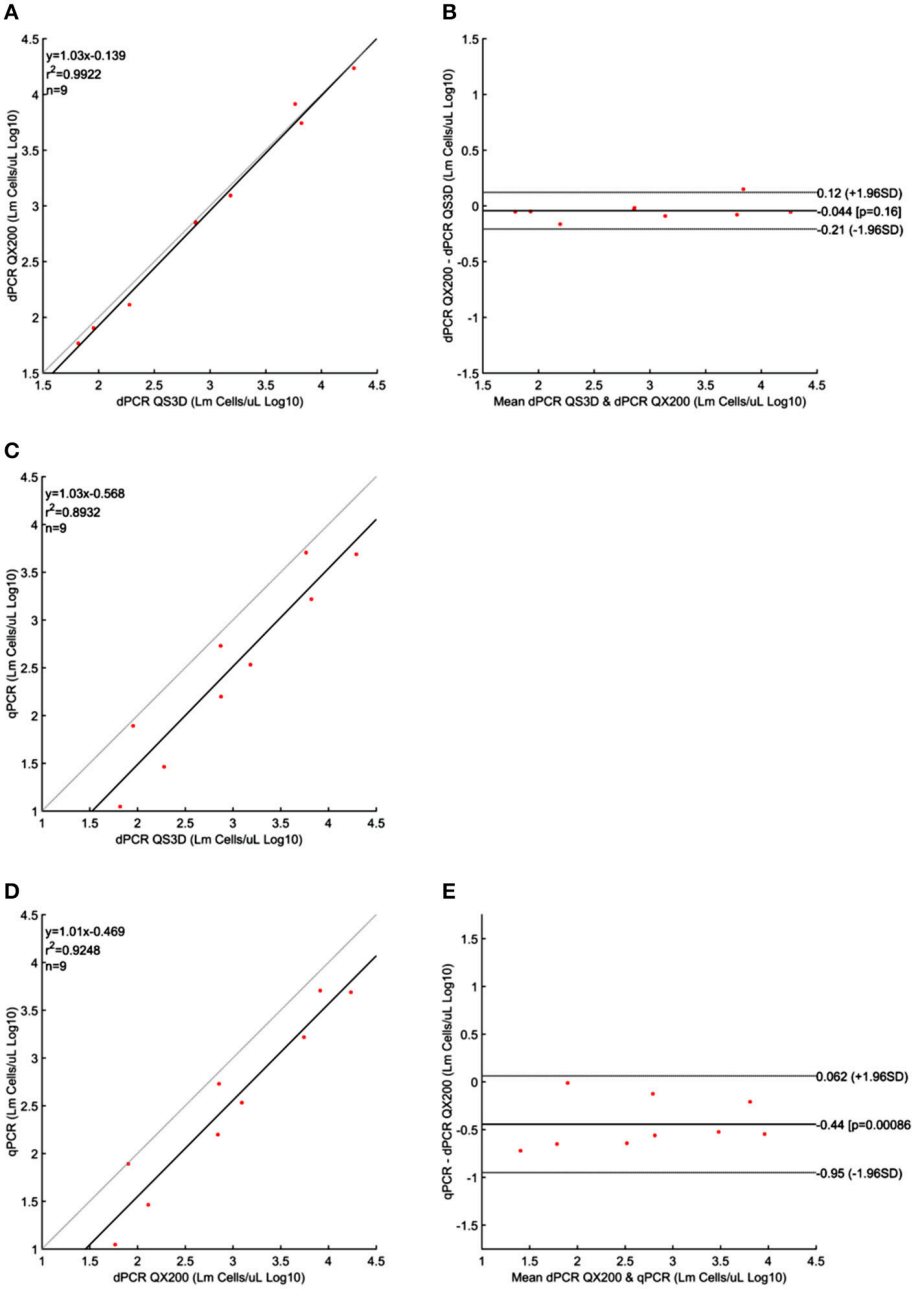
Linear regressions and Bland and Altman analyses of *L. monocytogenes* (Lm) cells in unknown samples obtained by dPCRs (QuantStudio 3D and QX200) and qPCR. **(A)** Linear regression between the two dPCRs; **(B)** Bland and Altman analysis between the two dPCRs; **(C)** Linear regression of dPCR QuantStudio 3D and qPCR; **(D)** Linear regression of dPCR QX200 and qPCR; **(E)** Bland and Altman analysis of dPCR QX200 and qPCR. Bland and Altman analysis between QuantStudio 3D and qPCR was not possible because the differences were not normally distributed. Data were reported as Log_10_ cells per μL. The gray lines in linear regression plots represent the ideal regression value.

Quantification results of *L. monocytogenes* are shown in Table 3. For the highest concentrations of *L. monocytogenes* cells (over 10^4^ cells per μL), both dPCR failed to quantify the number of bacteria, while the qPCR quantified up to 10^5^ cells per μL. In this case, after Log_10_ transformation, the difference between QS3D dPCR and qPCR was not normally distributed (*p* = 0.027) and it was not possible to make a comparison using Bland and Altman approach for this difference. The Log_10_-differences QS3D—QX200 and qPCR—QX200 were distributed normally with *p*-values 0.068 and 0.094, respectively. The interval, analyzed by Bland and Altman, was from 10^1^ to 10^3^–10^4^ *L. monocytogenes* cells, according to the LOQs of each PCR approach (see also Figure 1 and Table 2). The linear regressions showed *r*^2^-values between 0.893 and 0.992, while Bland and Altman analyses (Figure 3) exhibited all points falling inside the interval covered by the 95% CI (Figure 3B). The bias of agreement was approximately zero for the comparison between dPCRs (*p* = 0.160), while the comparison between qPCR and QX200 dPCR, showed a bias of −0.44 Log_10_ (*p* < 0.001). This result underlined how the qPCR quantification underestimated the number of *L. monocytogenes* cells in comparison to dPCRs. Notably, the intervals defined by the 95% CI for *L. monocytogenes* were narrower than those for MAP, and the zero value never fell within the intervals.

**Table 3:** Experimental output of *L. monocytogenes* cells obtained by hlyA-qPCR, hlyA—QX200 dPCR and hlyA—QuantStudio 3D dPCR.

		**qPCR^a^**			**dPCR QX200^b^**				**dPCR QuantStudio 3D^b^**			
	**Dilution**	**Mean**	**SD**	**Signal ratio^c^**	**Mean**	**SD**	**Mean λ**	**Signal ratio^c^**	**Mean**	**SD**	**Mean λ**	**Signal ratio^c^**
IZSLER 133/1/16	No dil	4.93 × 10^6^	1.35 × 10^6^	4/4	NT				NT			
	−1	5.33 × 10^5^	8.78 × 10^4^	4/4	NT				NT			
	−2	5.94 × 10^4^	1.55 × 10^3^	4/4	Saturated	Saturated	Saturated	2/2	Saturated	Saturated		2/2
	−3	4.86 × 10^3^	7.37 × 10^2^	4/4	1.72 × 10^4^	1.41 × 10^2^	3.65	2/2	1.95 × 10^4^	/		1/2
	−4	3.40 × 10^2^	4.80 × 10^1^	4/4	1.24 × 10^3^	5.94 × 10^1^	0.26	2/2	1.53 × 10^3^	1.15 × 10^2^	0.38	2/2
	−5	2.89 × 10^1^	2.07 × 10^0^	4/4	1.30 × 10^2^	1.98 × 10^0^	0.03	2/2	1.90 × 10^2^	2.53 × 10^0^	0.05	2/2
	−6	2.75 × 10^0^	9.59 × 10^−2^	4/4	1.26 × 10^1^	8.50 × 10^−1^	0.00	2/2	1.79 × 10^1^	5.70 × 10^−1^	0.00	2/2
IZSLER 129516/1/16	No dil	3.00 × 10^6^	1.26 × 10^5^	4/4	NT				NT			
	−1	2.31 × 10^5^	1.05 × 10^4^	4/4	NT				NT			
	−2	2.32 × 10^4^	2.98 × 10^3^	4/4	Saturated	Saturated	Saturated	2/2	Saturated	Saturated		2/2
	−3	1.65 × 10^3^	4.38 × 10^2^	4/4	5.53 × 10^3^	1.16 × 10^3^	1.17	2/2	6.63 × 10^3^	4.14 × 10^2^	1.67	2/2
	−4	1.58 × 10^2^	5.32 × 10^1^	4/4	6.94 × 10^2^	8.49 × 10^0^	0.15	2/2	7.48 × 10^2^	9.18 × 10^1^	0.19	2/2
	−5	1.11 × 10^1^	4.38 × 10^0^	4/4	5.84 × 10^1^	4.53 × 10^0^	0.01	2/2	6.58 × 10^1^	2.49 × 10^0^	0.02	2/2
	−6	1.07 × 10^0^	6.60 × 10^−1^	4/4	5.84 × 10^0^	1.92 × 10^0^	0.00	2/2	7.86 × 10^0^	6.90 × 10^−2^	0.00	2/2
IZSLER 424965/2/16	No dil	1.91 × 10^6^	3.19 × 10^5^	4/4	NT				NT			
	−1	2.18 × 10^5^	3.98 × 10^4^	4/4	NT				NT			
	−2	2.92 × 10^4^	3.55 × 10^3^	4/4	Saturated	Saturated	Saturated	2/2	Saturated	Saturated		2/2
	−3	2.16 × 10^3^	1.66 × 10^2^	4/4	8.20 × 10^3^	1.78 × 10^2^	1.74	2/2	5.82 × 10^3^	5.59 × 10^3^	1.46	2/2
	−4	1.52 × 10^2^	1.33 × 10^1^	4/4	7.14 × 10^2^	1.41 × 10^1^	0.15	2/2	7.43 × 10^2^	1.67 × 10^2^	0.19	2/2
	−5	1.45 × 10^1^	1.39 × 10−^1^	4/4	8.02 × 10^1^	5.37	0.02	2/2	9.0 × 10^1^	1.05 × 10^1^	0.02	2/2
	−6	1.2 × 10^0^	2.61 × 10−^1^	4/4	6.40 × 10^0^	0	0.00	2/2	1.3 × 10^1^	9.10 × 10^−1^	0.00	2/2
	−7	6.45 × 10^−2^	1.21 × 10^−2^	4/4	1.04 × 10^0^	6.80 × 10^−1^	0.00	2/2	1.44 × 10^0^	2.00 × 10^−1^	0.00	2/2

*NT, not tested*.

a *Number of L. monocytogenes cells/μL in the pure cultures/suspensions evaluated by hlyA-qPCR. Data are shown as mean and standard deviation of two qPCR runs in duplicated*.

b *Number of L. monocytogenes cells/μL in the pure cultures/suspensions evaluated by hlyA-dPCRs. Data are shown as mean and standard deviation of one dPCR runs in duplicated for both dPCR platforms*.

c *Number of positive or acceptable replicates/total number of replicates*.

#### Francisella tularensis

**Figure 4:**
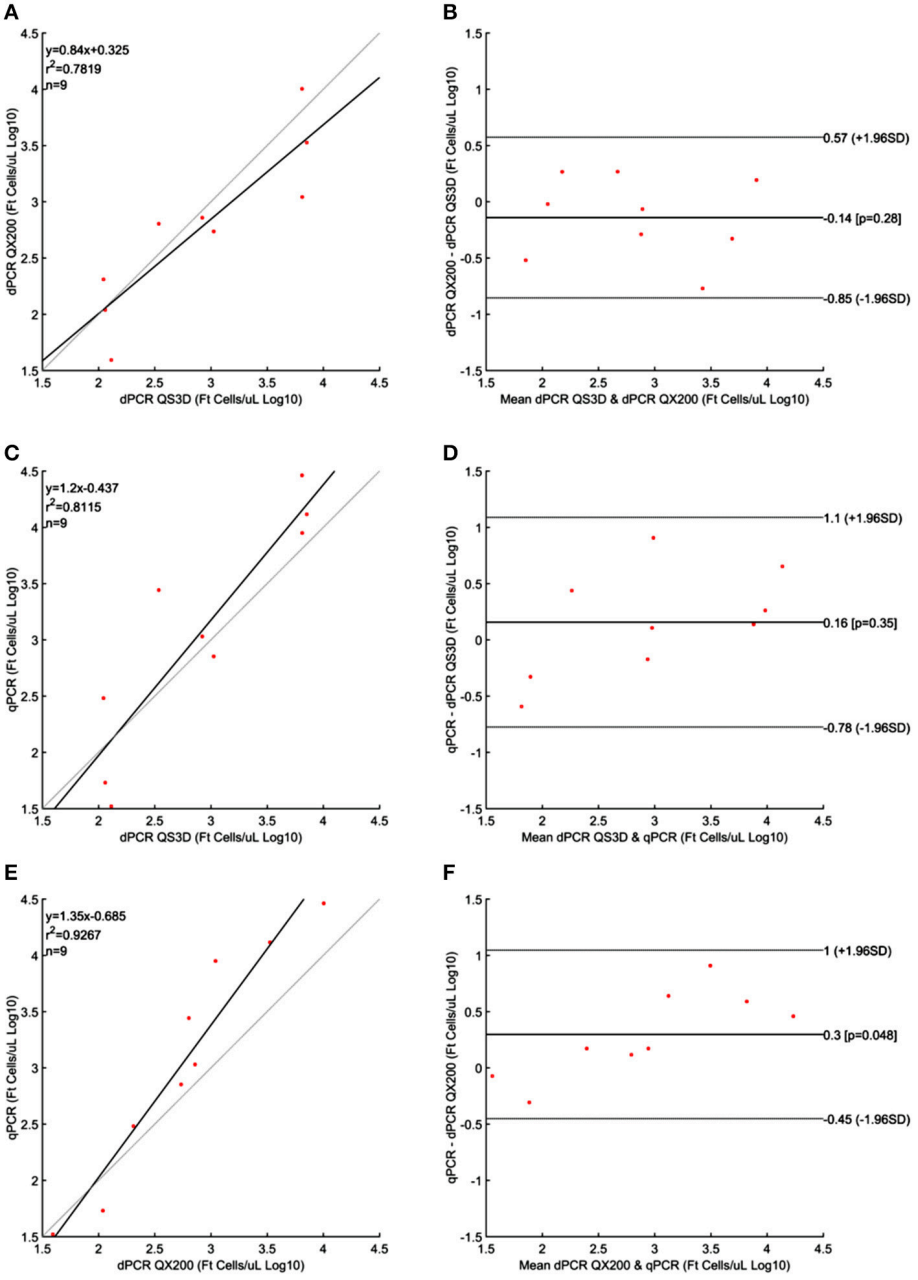
Linear regressions and Bland and Altman analyses of *F. tularensis* (Ft) cells in unknown samples obtained by dPCRs (QuantStudio 3D and QX200) and qPCR. **(A)** Linear regression between the two dPCRs; **(B)** Bland and Altman analysis between the two dPCRs; **(C)** Linear regression of dPCR QuantStudio 3D and qPCR; **(D)** Bland and Altman analysis of dPCR QuantStudio 3D and qPCR; **(E)** Linear regression of dPCR QX200 and qPCR; **(F)** Bland and Altman analysis of dPCR QX200 and qPCR. Data were reported as Log_10_ cells per μL. The gray lines in linear regression plots represent the ideal regression value.

The results obtained for *F. tularensis* are shown in Table 4 and Figure 4. Also in this case, at the highest concentrations of bacterium, both dPCR systems were saturated and it was not possible to quantify the number of cells present in the unknown suspensions (Table 4). For this reason, and according to the results shown in Table 1 and Figure 1 about the LOQ, the dilutions of unknown suspensions taken into account for the Bland and Altman analysis (Figure 4) were from 10^1^ to 10^3^. The Log_10_-differences among the three PCR approaches were distributed normally, with *p*-values of 0.505, 0.994 and 0.935 for QS3D—QX200, qPCR—QS3D and qPCR —QX200, respectively. The linear regressions showed *r*^2^-values between 0.782 and 0.927. Bland and Altman analysis (Figure 4) showed a bias for the agreement's range from −0.14 to 0.3 Log_10_ for all the comparisons, with the *zero* value falling within 95% CI of the agreement's limits. Overall, all PCR approaches quantified the same amount of bacteria, with the exception of the difference between qPCR—QX200 dPCR in which the *p*-value of the bias was 0.048. However, it should be noted that the limits of agreement are indeed wider than those previously obtained for MAP and *L. monocytogenes*, underlining a certain degree of variability in the quantification of *F. tularensis*.

**Table 4:** Experimental output of *F. tularensis* cells obtained by 23 kDa gene -qPCR, 23 kDa gene QX200-dPCR and 23 kDa gene-QuantStudio 3D dPCR.

		**qPCR^a^**			**dPCR QX200^b^**				**dPCR QuantStudio 3D^b^**			
	**Dilution**	**Mean**	**SD**	**Signal ratio^c^**	**Mean**	**SD**	**λ**	**Signal ratio^c^**	**Mean**	**SD**	**λ**	**Signal ratio^c^**
ATCC 6223	No dil	8.26 × 10^5^	1.27 × 10^5^	9/9	NT				NT			
	−1	1.10 × 10^5^	1.58 × 10^4^	9/9	Saturated	Saturated	Saturated		Saturated			
	−2	8.89 × 10^3^	2.45 × 10^3^	9/9	1.10 × 10^3^	1.99 × 10^2^	0.23	4/4	6.49 × 10^3^	1.82 × 10^3^	1.63	2/2
	−3	7.11 × 10^2^	2.62 × 10^2^	9/9	5.44 × 10^2^	1.37 × 10^2^	0.12	4/4	1.06 × 10^3^	1.24 × 10^1^	0.27	2/2
	−4	5.38 × 10^1^	2.30 × 10^1^	9/9	1.09 × 10^2^	1.46 × 10^1^	0.02	4/4	1.15 × 10^2^	6.46 × 10^0^	0.03	2/2
	−5	2.58 × 10^0^	1.56 × 10^0^	9/9	1.09 × 10^1^	4.12 × 10^0^	0.00	4/4	1.18 × 10^1^	/		1/2
	−6	2.10 × 10^−1^	8.64 × 10^−2^	9/9	2.19 × 10^0^	6.98 × 10^−1^	0.00	4/4	3.89 × 10^0^	/		1/2
IZSLER 42055/1/08	No dil	4.06 × 10^6^	1.51 × 10^6^	9/9	NT				NT			
	−1	5.09 × 10^5^	1.40 × 10^5^	9/9	Saturated	Saturated	Saturated		Saturated			
	−2	2.90 × 10^4^	7.00 × 10^3^	9/9	1.01 × 10^4^	5.15 × 10^3^	2.14	4/4	6.46 × 10^3^	1.66 × 10^2^	1.63	2/2
	−3	2.77 × 10^3^	1.27 × 10^3^	9/9	6.36 × 10^2^	4.89 × 10^2^	0.14	4/4	3.43 × 10^2^	2.85 × 10^1^	0.06	2/2
	−4	3.04 × 10^2^	3.84 × 10^1^	9/9	2.04 × 10^2^	1.21 × 10^2^	0.04	4/4	1.11 × 10^2^	5.66 × 10^0^	0.03	2/2
	−5	1.26 × 10^1^	5.10 × 10^0^	9/9	3.12 × 10^1^	1.16 × 10^1^	0.01	4/4	3.00 × 10^1^	2.67 × 10^0^	0.01	2/2
	−6	1.67 × 10^0^	1.08 × 10^0^	9/9	2.16 × 10^0^	6.75 × 10^−1^	0.00	4/4	2.11 × 10^0^	8.77 × 10^−1^	0.00	2/2
IZSLER 31895/1/09	No dil	5.77 × 10^6^	9.32 × 10^5^	9/9	NT				NT			
	−1	5.08 × 10^5^	3.54 × 10^4^	9/9	Saturated	Saturated	Saturated		Saturated			
	−2	1.30 × 10^4^	3.08 × 10^3^	9/9	6.70 × 10^3^	1.22 × 10^2^	1.42	2/2	7.13 × 10^3^	5.10 × 10^2^	1.79	2/2
	−3	1.07 × 10^3^	2.58 × 10^2^	9/9	1.44 × 10^3^	8.32 × 10^2^	0.31	2/2	8.39 × 10^2^	7.28 × 10^−1^	0.21	2/2
	−4	3.31 × 10^1^	8.17 × 10^0^	9/9	7.68 × 10^1^	1.13 × 10^0^	0.02	2/2	1.30 × 10^2^	3.42 × 10^0^	0.03	2/2
	−5	9.33 × 10^−1^	4.47 × 10^−1^	9/9	6.74 × 10^0^	4.04 × 10^0^	0.00	2/2	1.12 × 10^1^	1.94 × 10^0^	0.00	2/2
	−6	6.25 × 10^−2^	4.00 × 10^−2^	9/9	9.20 × 10^−1^	9.05 × 10^−1^	0.00	2/2	1.14 × 10^0^	1.27 × 10^0^	0.00	2/2

*NT, not tested*.

a *Number of F. tularensis cells/μL in the pure cultures/suspensions evaluated by 23 kDa gene-qPCR. Data are shown as mean and standard deviation of three qPCR runs in triplicate*.

b *Number of F. tularensis cells/μL in the pure cultures/suspensions evaluated by 23 kDa gene -dPCRs. Data are shown as mean and standard deviation of two dPCR runs in duplicated for QX200 dPCR and one dPCR run in duplicate for QuantStudio 3D dPCR (with the exception of strain IZSLER 31895/1/09 in which also QX200 dPCR was run only once in duplicate*.

c *Number of positive or acceptable replicates/total number of replicates*.

### Evaluation of the number of bacteria in unknown suspensions by other methods

The results regarding the quantification obtained with “non-molecular methods” are shown in Table 5. For MAP, the direct counting by Burker chamber underestimated the number of bacterial cells for approximately one Log_10_ than the quantification done by PCR approaches, this difference rose up to two Log_10_ for plate counting method. *F. tularensis* showed similar results for plate counting, while for *L. monocytogenes* there were no differences. This last result allowed the assessment of the reproducibility for the quantification of *L. monocyotogenes* obtained by both PCR approaches and cultural assay.

**Table 5:** Quantification of the unknown water suspensions carried out by PCR approaches and non-molecular methods.

	***Mycobacterium avium*** **subsp** ***paratuberculosis***			***Listeria monocytogenes***			***Francisella tularensis***		
**Strain**	**ATCC 19698**	**IZSLER 623/15**	**IZSLER 22/16**	**IZSLER 133/1/16**	**IZSLER 129516/1/16**	**IZSLER 424965/2/16**	**ATCC 6223**	**IZSLER 42055/08**	**IZSLER 318595/09**
Direct count^a^	3.7 × 10^4^	3.6 × 10^4^	3.5 × 10^4^						
Absorbance^b^	0.06	0.09	0.04	1.54	1.17	1.25	0.86	0.84	0.77
Plate counting^c^	1.3 × 10^3^	1.4 × 10^3^	1.5 × 10^3^	1.0 × 10^7^	2.5 × 10^6^	3.1 × 10^6^	3.7 × 10^4^	4.2 × 10^4^	7.5 × 10^4^
qPCR^d^	3.6 × 10^5^	5.0 × 10^5^	2.2 × 10^5^	4.9 × 10^6^	3.0 × 10^6^	1.9 × 10^6^	8.3 × 10^5^	4.0 × 10^6^	5.8 × 10^6^
QX200^e^	1.3 × 10^5^	2.2 × 10^5^	1.6 × 10^5^	1.7 × 10^7^	5.5 × 10^6^	8.2 × 10^6^	1.1 × 10^5^	1.0 × 10^6^	6.7 × 10^5^
QS3D^e^	5.0 × 10^4^	2.2 × 10^5^	1.3 × 10^5^	1.9 × 10^7^	6.6 × 10^6^	5.8 × 10^6^	6.5 × 10^5^	6.5 × 10^5^	7.1 × 10^5^

*The results are referred to the initial unknown suspensions before dilution*.

a *Number of MAP cells/μL evaluated by Burker chamber, counting 20 squares and calculating the final result with the web tool available at the web site of “The Ebert Group” (http://evolution.unibas.ch/ebert/lab/counting.htm)*.

b *Absorbance recorded at 600 nm*.

c *Number of bacteria in the unknown water suspensions evaluated by cultural plating in appropriate medium, for more details refer to material and method section. Results are expressed as CFU/μL*.

d *For MAP, it was evaluated by multiplying per ten the number of bacteria recovered at the dilution −1. Results are expressed as number of cells/μL*.

e *Evaluated by multiplying for the Log_10_ factor from the first dilution in which it was possible to count the number of bacteria Results are expressed as number of cells/μL*.

### Evaluation of the level of uncertainty for *L. monocytogenes* associated with the methods

The levels of uncertainty for *L. monocyotogenes* for each method is shown in Table 6. The uncertainty associated with PCR methods was similar or lower to that associated with the cultural assay.

**Table 6:** Level of uncertainty relative to *L. monocytogenes* for all the methods.

	**Uncertainty**
Plate counting	0.208
qPCR	0.062
QX200	0.113
QS3D	0.167

*Data were Log_10_ transformed before uncertainty's evaluation according to the Material and Method section*.

## Discussion

The aim of our work was to evaluate if the quantifications obtained by qPCR were comparable to those obtained by two different dPCR systems. Additionally, a comparison among PCR approaches and non-molecular methods was also evaluated.

All the bacteria analyzed in the study are human or animal pathogens and their detection, and quantification, for diagnostic purposes, is relevant, such as during the development of risk exposure analysis models. In these models, the quantification of the real amount of bacteria in a given sample plays a crucial role in the definition of risk (Halder et al., 2010). Therefore, the interest in developing faster and reliable quantitative methods for detection and quantification is becoming a very important issue in microbiology. This is particularly relevant for some pathogens characterized by slow cultural growth rates (e.g., MAP), but generally, rapidity can be advantageous for detection and quantification of all pathogens. In this regard, some risk models have already been proposed for MAP (Nauta and van der Giessen, 1998; Boulais et al., 2011), *F. tularensis* (Wood et al., 2014) and *L. monocyotogenes* (Halder et al., 2010).

According to the Minimum Information for Publication of Quantitative Real-Time PCR experiments (Bustin et al., 2009) and the Minimum Information for Publication of Quantitative Digital PCR Experiments (Huggett et al., 2013) guidelines we provided here data about the performances of the qPCRs and dPCRs used in the study and aimed at quantifying of MAP, *L. monocytogenes* and *F. tularensis*. In order to assess if primers and probes can work in the same way for both qPCR and dPCR, the LOD, LOQ, and the other performance parameters of all dPCRs were done without any further specific reaction optimization and compared to those observed for qPCRs.

Comparison between qPCR and dPCR quantification was performed on a panel of bacterial suspension samples. During the estimation of the titres in the unknown samples, Bland and Altman approach was carried out considering only a few of the tested samples. In fact, it was not possible to include more points in these analyses because: (i) for qPCRs, the limits of quantification (coefficient of variation <25%) did not permit the inclusion of the lowest concentrations, (ii) the dPCR systems were saturated at the highest concentrations. So the available range to perform the analyses was restricted from one to three-four Log_10_ cells per μL.

Despite the aforementioned limits, the linear regressions and the Bland and Altman analyses (these last when the differences in Log_10_ transformed data were normally distributed) for both dPCR platforms, showed a strong correlation (*r*^2^-values 0.961, 0.992, and 0.782 for MAP, *L. monocyotogenes* and *F. tularensis*, respectively). Moreover, both systems quantified a very similar amount of bacteria. However, the analysis of the differences in quantification among qPCR and dPCRs showed that f57-qPCR over estimated, by a 0.3 Log_10_, the number of MAP cells respect to both the dPCR platforms (*p*-values 0.06 and 0.03; Figures 2C,E). The overestimation effect on the quantification of microorganisms by qPCR was also recently observed for in the quantification of *M. tuberculosis* (Devonshire et al., 2016) and others works addressed to the quantification of virus (Boizeau et al., 2014; Gosselin-Theberge et al., 2016; Supplementary Material 2). Similarly, also 23 kDa gene-qPCR for the quantification of *F. tularensis* over estimated by 0.3 Log_10_ the number of cells (*p*-value 0.048), (Figure 4E), but only considering the difference between qPCR and QX200, while between qPCR and QS3D no difference was observed (0.16 Log_10_ but *p* = 0.35).

On the other hand, other papers reported a general agreement in the amount of target NAs quantified by qPCR and dPCR, both for virus (Hayden et al., 2013; Pavšič et al., 2016) as well as for bacterial quantifications (Verhaegen et al., 2016; Wang et al., 2016; Witte et al., 2016).

Finally, the comparison between the QX200 platform and qPCR for *L. monocytogenes*, showed qPCR can underestimate the number of cells by 0.44 Log_10_ (*p* < 0.001) and, according to the linear regression curve (Figure 3C), also the comparison between qPCR and QS3D dPCR showed an underestimation of the cells number by qPCR. In this regard, two recent papers reported similar results: one was addressed to the quantification poultry pathogens (Rothrock et al., 2013) and the other to the quantification of *Campylobacter jejuni* (Papic et al., 2017). A table summarizing these observations is reported in Data Sheet 2 (Supplementary Material 2).

Digital PCR generally is accredited to provide more accurate measurements than qPCR, because it is less affected by the presence of inhibitors and by poor amplification efficiency than qPCR (Hoshino and Inagaki, 2012; Hudecova, 2015; Pavšič et al., 2015); moreover it is not affected at all by the requirement of NA standards. In fact, it gives an absolute estimate of concentration, through the partition of the sample in template individual reactions. Therefore, dPCR does not need any standards for the generation of calibration curves.

Despite its independence on standard curves, compared to qPCR, dPCR is more time consuming and labor intensive. Nevertheless, excluding the initial fee for acquiring the system and considering a run section of 50 samples, the cost of the analysis was less for dPCR (QX200; 3.16 € per sample) than for qPCR (10.80 € per sample).

Some limitations are also present in dPCR systems. In our study, as above mentioned, when pure genomic standards DNA were used at the highest concentration, both dPCR systems were saturated and it was not possible to quantify the number of bacteria. The dynamic range of dPCRs was limited by the number of available partitions, which were around 20,000 for both QX200 and QSD3, while the qPCRs showed higher dynamic ranges.

The comparison between quantification by PCR approaches and cultural methods showed some differences, which appear associated to the specific time of growth for each microorganism. In particular, MAP required a very long time to grow (at least 4 weeks) and our results confirmed previous observations in which there is a difference from one to two Log_10_ between plate counting method and PCRs (Herthnek et al., 2008; Kralik et al., 2012; Table 5). Instead, the difference between PCRs and direct counting method was about one Log_10_. This could be particularly relevant, because some papers used the direct count method to determine the number of MAP cells during the assessment of LOD for PCR assays (Tasara and Stephan, 2005; Plain et al., 2014).

Comparison between plate counting and PCRs for *F. tularensis*, which has a time of growth in culture of 4–5 days, showed results similar to those obtained for MAP, but the difference between the two methods was less evident. In order to avoid any bias in the determination of the LOD during the PCR assay validation, this difference should be taken into account (Versage et al., 2003). In fact, to bypass this problem, the most recently developed methods have used genomic pure DNA or plasmid standards instead of pure cultures of *Francisella* (Euler et al., 2012; Seiner et al., 2013).

On the contrary, no differences were found for *L. monocyotogenes* (24 h of growth in blood agar), between plate counting and PCRs. This suggests the possibility to quantify *L. monocyotogenes* with molecular assays, which seems to be a reliable alternative to the cultural method. In this context, for *L. monocytogenes*, a quantitative maximum limit of contamination, is stated by the Reg EC 2073/2005, which in turn defines the microbiological criteria for foodstuffs (Anonymous, 2005). The level defined for *L. monocytogenes* is “≤100 CFU/g for Ready To Eat (RTE) foodstuffs.” Notably, the analytical time for traditional microbiological methods varies from 2 to 3 days, but, it can even take up to a week before a final result is achieved for positive samples. This could be a serious problem for the commercialization of RTE products, because often they have a short shelf–life.

In addition, since *L. monocytogenes* is the only pathogen in which a quantitative maximum limit has been defined (100 CFU/g), it is important to express the uncertainty associated with the use of PCR approaches and compare it to that for cultural assay (Anonymous, 2009). Our results suggest the level of uncertainty of both qPCR and dPCRs was similar or lower to that calculated for the cultural assay (Table 6). This result clearly demonstrated how PCR approaches are suitable for the quantification and can represent a valid alternative for the enumeration of *L. monocytogenes*.

In conclusion, the quantifications of MAP, *L. monocytogenes* and *F. tularensis* by the two dPCR platforms were highly comparable to each other. However, the comparison between qPCR and dPCR suggested some differences are present. Probably, it is not always possible to pass between qPCR and dPCR without any optimization of the concentration of primers and probes. One of the critical points for the enumeration by qPCR can be the difficulty in the quantification of the standards needed for the building of the calibration curves. This issue represents a challenging factor for the standardization of a quantifying method and also implies a higher reagents cost. Notably, the request of requirements for international standards for molecular biology assays is increasing and, since the production of useful molecular biology reference materials poses some technical challenges, one European project, the “Metrology for Monitoring Infectious Diseases, Antimicrobial Resistance and Harmful Micro-organisms,” also addressed this purpose (Pavšič et al., 2015). If more reference material becomes available, we believe PCRs approaches can became the new gold standard methods for the quantification of bacterial pathogens.

## Author contributions

MR, MBB, NV, and BB conceived, designed and wrote the paper. CB helped to write the paper and performed the experiments with QX200 dPCR. SR performed the quantification by qPCR and other methods for MAP. MT performed the experiment about *L. monocytogenes* and all experiment with Quant Studio 3D dPCR. NV and MAB performed the quantification by qPCR and other methods for *F. tularensis*.

### Conflict of interest statement

The authors declare that the research was conducted in the absence of any commercial or financial relationships that could be construed as a potential conflict of interest.
